# Cancer registration by linking pathology and district PAS data.

**DOI:** 10.1038/bjc.1990.275

**Published:** 1990-08

**Authors:** B. W. Codling, D. Pheby, D. L. Hagen, M. F. Duffin

**Affiliations:** Gloucestershire Royal Hospital, Gloucester, UK.

## Abstract

The incompleteness and inaccuracy of cancer registries, with the resulting underestimation of cancer incidence and lack of confidence by clinicians in the information offered, have been noted by numerous studies throughout the world. We report attempts in one district to provide more accurate and timely information on cancer cases, both for local purposes and for expediting input to the Regional Registry and hence the National Registry. A semi-automatic link between the pathology system and the PAS system was developed to establish a histopathology-based dataset of cancer cases for the district. This software provides a basis for cancer registration and, combined with clinical staging and treatment ranges, could provide a timely and accurate picture of cancer for research, management, treatment and planning purposes.


Br. J. Cancer (1990), 62, 271-274 © Macmillan Press Ltd., 1990

### Cancer registration by linking pathology and District PAS data

B.W. Codling[1], D. Pheby[2], D.L. Hagen[2] & M.F. Duffin[1]

[1]Gloucestershire Royal Hospital, Great Western Road, Gloucester, UK; and [2]Gloucester Health Authority, Rikenel, Montpellier, Gloucester, UK.

**Summary** The incompleteness and inaccuracy of cancer registries, with the resulting underestimation of cancer incidence and lack of confidence by clinicians in the information offered, have been noted by numerous studies throughout the world. We report attempts in one district to provide more accurate and timely information on cancer cases, both for local purposes and for expediting input to the Regional Registry and hence the National Registry. A semi-automatic link between the pathology system and the PAS system was developed to establish a histopathology-based dataset of cancer cases for the district. This software provides a basis for cancer registration and, combined with clinical staging and treatment ranges, could provide a timely and accurate picture of cancer for research, management, treatment and planning purposes.

There is no question that to provide reliable, complete and timely data on which to base District and hence Regional Cancer Registration is vital (Nwene & Smith, 1982). In attempting to answer enquiries at a local level from clinicians, epidemiologists and researchers about the occurrence of neoplastic disease in the District, it was clear not only that the existing cancer registry was in many cases unable to supply the information required, but also that much of the information needed was to be found in District-based computer systems, but in unlinked form. However, a major impediment was an apparent inability to link PAS data containing patient identifications details with Pathology data which identified the neoplasm and site.

Others have noted the value of using a histopathology-based system to improve the completeness and accuracy of cancer registration. (Donnan *et al.*, 1981). This would have the advantage of enabling details to be obtained of patients treated by private clinics, private hospitals and general practitioners. In addition, this would reduce the inaccuracy rates in site and histology coding, as up to ten per cent are transcription errors caused by CDC clerks in coding (West 1976; Lockwood 1971). Even higher figures are noted in more recent studies, including, for example, the Oxford FPA contraceptive study, in which there was a 2.5 year time delay in communication with the NHS Central Registry at Southport (Villard-Mackintosh *et al.*, 1988). Other advantages include improvements in timeliness which would enable more complete analysis, as well as the innate increase in completeness inherent in data extraction from operational systems. The Pathology Department computer is the obvious choice for the initiating step in the process of compiling a cancer registry.

The Pathology Department system is based on the designation of a SNOMED code which is a specific morphological and topographical identification applied to the tumour. In the normal process of cancer registration, the information on the disease is attached to the patient identification data as an ICD-9 code by the CDC/cancer registration clerk, often with little training, and often from illegible, or inadequate, clinical information on the HMR1 form (Donan *et al.*, 1981). The information reaches the CDC clerk by a roundabout route, being supplied indirectly by the pathologist to the clinician on report forms, the latter in turn filling out the HMR1 on death or discharge.

It was recognised that, if this link could be semi-automated to merge the pathology data into district PAS data, the accuracy and efficiency at this vital step could be vastly improved, the flow of information made much more direct, and human error eliminated. To this end, in Gloucester a link was developed between the pathology database and the PAS database by using the patient registration number held in common by both systems, and applying an algorithm which converts the SNOMED code to the appropriate ICD9 code.

Correspondence: B.W. Codling.
Received 15 January 1990; and in revised form 2 April 1990.

### Methods

Initially communication and enquiry programs from within the Pathology Department were established with the PAS computer which enabled patient details to be linked with histological results. This was done via the patient registration number and enabled local research and retrieval projects to be carried out, including validation of data within the histopathology data base. This program could be easily altered, if necessary, to search on more fields, for examples, names, forename and date of birth.

The next task was to establish a more direct means of providing data to the Regional Cancer Registry. Consistency was sought to provide data which conformed with Regional requirements. Direct provision of data to the Regional Registry would require translation of the SNOMED pathology code to the ICD9 code used by CDC and the Cancer Registry. The District Department of Public Health provided the initial algorithm for allocating ICD9 codes to the range of histopathology specimen data available on the Pathology Department computer. Outside assistance by means of research funds provided the programming needs for the algorithm. Data entry facilities were developed to update and alter the range of diagnoses which can be translated.

In developing the algorithm, the benign neoplasms were branched off early by a conditional step and then allocated via a specific schedule. Separate schedules are used early in processing for malignant melanomas, sarcomas (both bone and other sites) and hydatidiform moles because of different topography codes. The main group of *in situ* and malignant neoplasms are then sorted first by malignancy code, then in separate schedules for allocation purposes. (Figure 1). Initially the translation algorithm had been developed on the basis of data relating to one year's clinical activity in another health district but the algorithm was designed in such a way as to enable it to be updated and grow as new neoplasms were encountered whilst the software package was in use.

The code translations from SNOMED to ICD9 are made at the time of combining the histology and PAS data and are temporarily stored along with other relevant data in a workfile where they are available for rapid review and correction as necessary. After correction, overnight interrogation is performed and followed by rapid printing and reviewing. The present system is flexible, consisting of a small base program with several separate schedules. This allows conditional branches to be created when necessary and home updating is a relatively easy task.

**Figure 1** Algorithm of code translation from SNOMED to ICD9.

### Results

The system has been in use in its present form in this district for over a year and several observations can be made. Initially, the most valuable by-product of the system was its ability to validate and verify the SNOMED coding performed in the Pathology Department to a certain degree. If the program cannot allocate an ICD9 code, the computer will tag the specimen data on the workfile and enable rapid reviewing to correct inaccuracies and omissions in the schedules. Use has expanded the dataset and additional allocation steps were created when required. Now the stage has been reached where unallocated codes are at a satisfactorily low and steadily diminishing level.

Table I shows the results of print-outs for specimens reported in January and July 1989. It shows 'addresses not found' were minimal in the central district area and those that were noted consisted of patients not yet within PAS from out-patient attendances. In-patient searches yield only one or two unmatched addresses per month. A manual search of the FPC database produced a 95 per cent success rate in locating patient identification details.

In a similar manner, coding from SNOMED to ICD9 was manually validated for the same two months. Of the 279 specimens in January, 157 were malignant tumours and of those only six could not be allocated on ICD9 code by the program. In July 218 specimens were reported, 124 of which were malignant and two were not allocated. At those times the schedule for benign tumours was not operational hence the higher percentage of 'misses' noted for benign neoplasms in the table. A 'skip' in these terms means that the neoplasm was successfully identified as benign and therefore not required for cancer registration purposes. Of the 82 benign neoplasms in January, 12 were not allocated ICD9 codes; in July, of the 94 benign tumours, 16 were not allocated. This situation has since been rectified and better results are now being obtained after correction of clerical errors and missing codes within the algorithm.

**Table I** Results of searches, January and July 1989

| | *January* | *July* |
|---|---|---|
| **Address not found** | | |
| General practice | 32 (13.5%) | 35 (16%) |
| Private patients | 7 (3%) | 15 (6.9%) |
| Hospital | 10 (4%) | 5 (2.3%) |
| Total | 49 | 55 |
| **Coding from SNOMED to ICD 9** | | |
| Carcinoma, coded | 151 | 122 |
| Carcinoma, not allocated | 6 | 2 |
| Benign skip | 70 | 78 |
| Benign not allocated | 12 | 16 |
| Total | 239 | 218 |

The print-out designated for CDC and hence the Cancer Registry is shown in Figure 2. It contains the ICD9 code replacing the SNOMED code as well as patient identification data. If there is a message 'No code', the neoplasm was not allocated and hence must be processed manually, or corrected, and the program run again. By amending the appropriate schedule, the program can recognise the code on future runs. The data base print-out can be expanded to include such data as unique district identification number, NHS number and the residents' postcode, but currently this is not necessary in this district.

The system will detect obvious miscoding if the

```

LISTING FOR TOPOGRAPHY:  ALL  MORPHOLOGY: 8.. & 9..   27-Mar-90  10:52 AM

FROM  19/08/88  TO  19/08/88

Hospital No.  DATE        Surname:First Names      Date of Birth
* (Extra Search Data Used If Not Found From HOSP.No.)
PATIENT's ADDRESS
Specimen No.  Hospital Ward  CONSULT./GP PATHOLOGIST  CODED DIAGNOSES

00906231  19/08/88  J-----N:M----L E   13/02/1929

ROSE COTTAGE  B-------K,  N-----M,  GLOS.

H8808194  GRH  10  MR H THOMSON  DR N A SHEPHERD   1749;85203

00775559  19/08/88  H---Y:C--A S   31/10/1909

-1, D------E C---T,  WOTTON-UNDER-EDGE,  GLOS.

H8808195  GRH  SOPD  MR H THOMSON  DR N A SHEPHERD   1541;81403

00273668  19/08/88  W---E:A---E   19/07/1916

THE B-------W  THE G---S  E--------N  STONEHOUSE

H8808205  GRH  16  MR.C.CRAWSHAW  DR N A SHEPHERD   NoCode 11712;85005

00453621  19/08/88  K--------R:R-----D C   15/04/1915
17 K------- R  A----E  GLOUCESTER
H8808208  GRH  10  MR H THOMSON  DR N A SHEPHERD   NoCode 68000;8140

H0014716  19/08/88  R-- E:F------E   06/09/1911
NO ADDRESS -SEARCH3
H8808215  9030   DR R J ROWLANDS  DR N A SHEPHERD   2327;80812

00842525  19/08/88  W------Y:E----N G   08/10/1928

41 V------A D---E  E--------N  S---------E  GLOS

H8808220  STRO  STOP  MR G SWINGLER  DR N A SHEPHERD   2331;81402

00442602  19/08/88  L-------- Y:W-----E J   27/07/1922

3 P--T O----E C------S   L-----Y  GLOUCESTER

H8808223  GRH  EC  MR J O KILBY  DR.J.S.UFF   1509;81403

RECORDS FOUND : 13
RECORDS SKIPPED (BENIGN) : 6   RECORDS SHOWN : 7
No SNOMED to ICD-9 Translation found on : 2 codes.
Address found on Search1 = 9, Search2 = 2, Search3 = 0, Not Found = 2
```

**Figure 2** Database printout for CDC.

deliminators are not in the correct place. If there are not enough digits, or an inappropriate digit, in a field the algorithm cannot translate the codes, therefore the program will alert the users. Translating appropriate miscodes cannot be detected by this method but these are usually detected by the pathologists when they issue the reports, or by the CDC clerical officer who matches the print-out provided with other data from different sources. Some tumours are clinically diagnosed but they do not have histology confirmation. These tumours which are few in number, are spotted by the CDC staff.

### Discussion

Owing to failure to provide timely and accurate data for local epidemiological and clinical audit purposes, a data base was developed in this district based on histopathological results. To expedite the vital step at linking the histopathology results with patient information, a system operates using an algorithm to translate SNOMED to ICD9 coding and then print out this code with other patient identification details for use by the CDC/Cancer Registration clerks. From the outset the system was useful for validation and verification and has since proved its usefulness for local retrieval purposes. Although still being refined, the system already has a good success rate at locating patient information details and for allocating codes.

Creating this vital semi-automatic link has improved the accuracy and speed of reporting to the Regional Cancer Registry. In addition, it enables local efforts at obtaining information for use by epidemiologists and clinicians possible by establishing a local database of cancer patient information.

Access to the Family Practitioner Committee data base would greatly enhance the patient identification information available to match to the pathology data. At present this must be performed manually for individuals not within the PAS database. Private patients could be located from within the FPC database.

Efforts are now underway to incorporate fields recording clinical staging into the data base by means of an algorithm being developed within the District (Pheby 1989). In addition, information on treatment modalities will be added. When these efforts are completed, there will be available for the first time, accurate and timely clinical information for peer review, epidemiological research, the planning and evaluation of services and the management of resources (South Western Regional Health Authority 1989).

### References

DONNAN, S., BAGENAL, F. & ROBINSON, D. (1981). Cancer registration in Wessex, the value of the current system and possibilities for the future. *Department of Community Medicine, University of Southampton.*

LOCKWOOD, E. (1971). Accuracy of Scottish hospital morbidity data. *Br. J. Prev. Soc. Med.*, **25**, 76.

NWENE, V. & SMITH, A. (1982). Assessing completeness of cancer registration in the North-Western Region of England by a method of independent comparison. *Br. J. Cancer*, **46**, 635.

PHEBY, D. (1989). On the feasibility of incorporating data on clinical staging into the cancer registry dataset. *South Western Regional Health Authority.*

VILLARD-MACKINTOSH, L., COLEMAN, M.P. & VESSEY, M.P. (1988). The completeness of cancer registration in England, an assessment from the Oxford FPA contraceptive study. *Br. J. Cancer*, **58**, 507.

WEST, R.R. (1976). Accuracy of cancer registration. *Br. J. Prev. Soc. Med.*, **30**, 187.

Better patient information (1989). *South Western Regional Health Authority.*


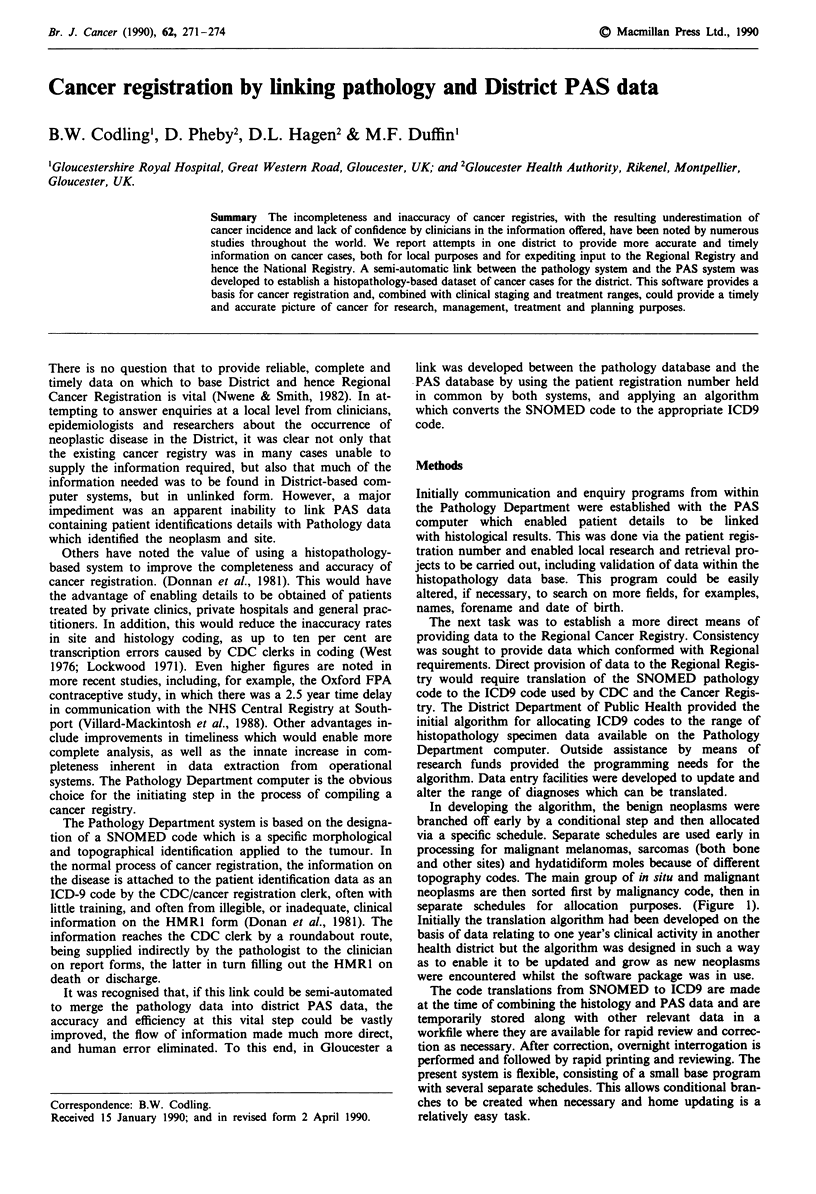

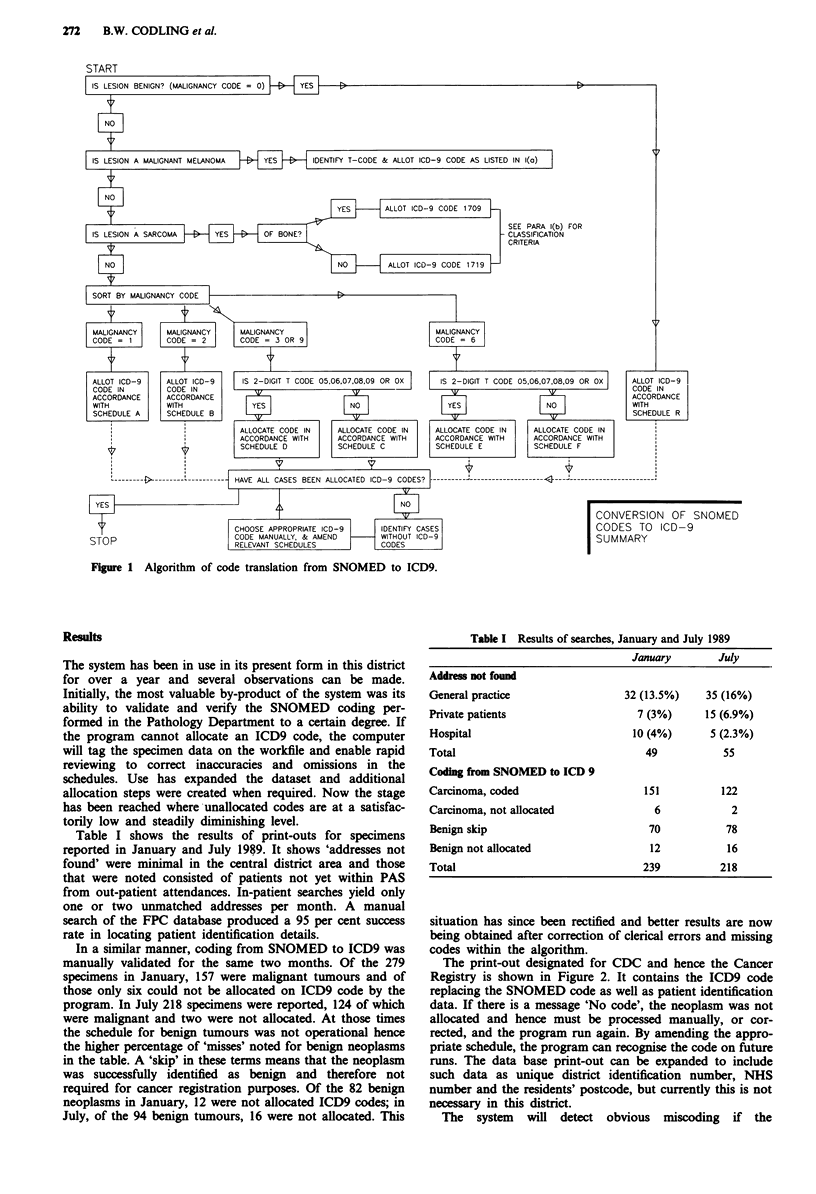

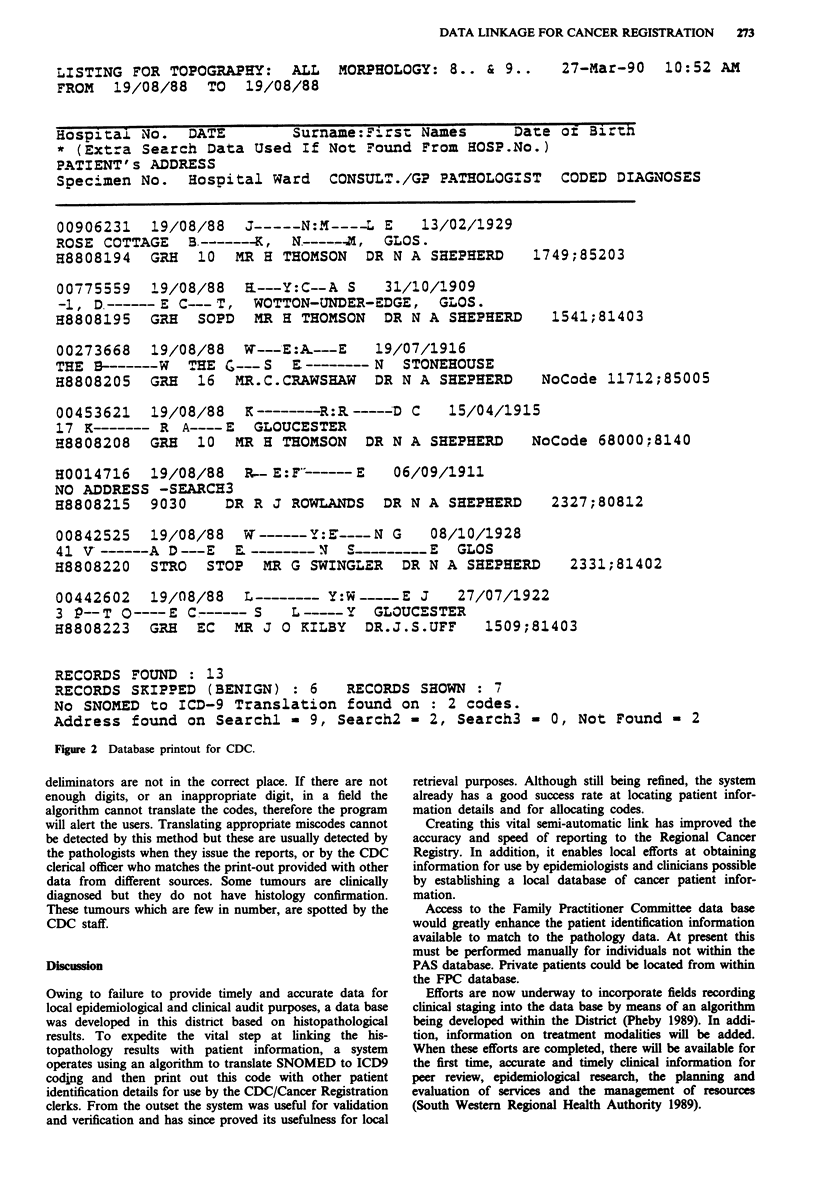

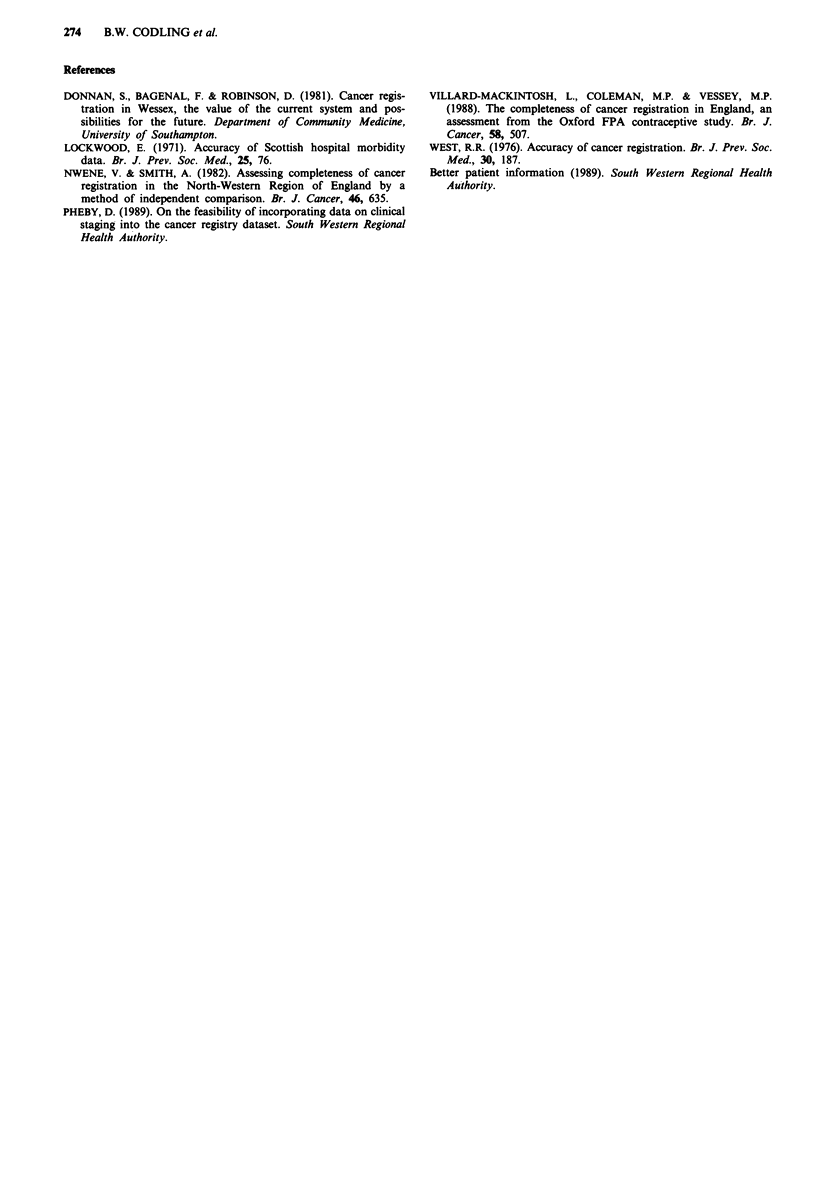


## References

[OCR_00393] Lockwood E. (1971). Accuracy of Scottish hospital morbidity data.. Br J Prev Soc Med.

[OCR_00397] Nwene U., Smith A. (1982). Assessing completeness of cancer registration in the north-western region of England by a method of independent comparison.. Br J Cancer.

[OCR_00407] Villard-Mackintosh L., Coleman M. P., Vessey M. P. (1988). The completeness of cancer registration in England: an assessment from the Oxford-FPA contraceptive study.. Br J Cancer.

[OCR_00413] West R. R. (1976). Accuracy of cancer registration.. Br J Prev Soc Med.

